# Supplementary material for: A renewed rise in global HCFC-141b emissions between 2017–2021
Source: Atmos Chem Phys. Author manuscript; Available in PMC 2024 Sep 23. (PMC11417968; doi:10.5194/acp-22-9601-2022)
Supplement: Supplement [file NIHMS1957572-supplement-Supplement.pdf]

A rise in global HCFC-141b emissions between 2017-2021

Luke M. Western[1,2], Alison L. Redington[3], Alistair J. Manning[3], Cathy M. Trudinger[4], Lei Hu[1,5], Stephan Henne[6], Xuekun Fang[7], Lambert J.M. Kuijpers[8], Christina Theodoridi[9], David S. Godwin[10], Jgor Arduini[11], Bronwyn Dunse[4], Andreas Engel[12], Paul J. Fraser[4], Christina M. Harth[13], Paul B. Krummel[4], Michela Maione[11], Jens Mühle[13], Simon O'Doherty[2], Hyeri Park[14], Sunyoung Park[14], Stefan Reimann[6], Peter K. Salameh[13], Daniel Say[2], Roland Schmidt[13], Tanja Schuck[12], Carolina Siso[1,5], Kieran M. Stanley[12], Isaac Vimont[1,5], Martin K. Vollmer[6], Dickon Young[2], Ronald G. Prinn[15], Ray F. Weiss[13], Stephen A. Montzka[1], and Matthew Rigby[2]

[1]Global Monitoring Laboratory, National Oceanic and Atmospheric Administration, Boulder, CO, USA
[2]School of Chemistry, University of Bristol, Bristol, UK
[3]Hadley Centre, Met Office, Exeter, UK
[4]Climate Science Centre, CSIRO Oceans and Atmosphere, Aspendale, Victoria, Australia
[5]Cooperative Institute for Research in Environmental Sciences, University of Colorado, University of Colorado, USA
[6]Empa, Swiss Federal Laboratories for Materials Science and Technology, Dübendorf, Switzerland
[7]College of Environmental and Resource Sciences, Zhejiang University, China
[8]A/gent b.v. Consultancy, Venlo, Netherlands
[9]Natural Resources Defense Council, USA
[10]Stratospheric Protection Division, Environmental Protection Agency, Washington, DC, USA
[11]Department of Pure and Applied Sciences, University of Urbino, Urbino, Italy
[12]Institute for Atmospheric and Environmental Science, Goethe University Frankfurt, Frankfurt am Main, Germany
[13]Scripps Institution of Oceanography, University of California San Diego, La Jolla, CA, USA
[14]Department of Oceanography, Kyungpook National University, Daegu, Republic of Korea
[15]Center for Global Change Science, Massachusetts Institute of Technology, Cambridge, MA, USA

Correspondence: Luke M. Western (luke.western@noaa.gov/luke.western@bristol.ac.uk)

Station	Country	Longitude & Latitude	Instrument	Data period
		NOAA		
South Pole (SPO)	Antarctica	90 °S	GCMS	1993/01 - 2021/12
Cape Grim (CGO)	Australia	41 °S, 145 °E	GCMS	1992/12 - 2021/12
Cape Matatula (SMO)	America Samoa	14 °S, 171 °W	GCMS	1993/01 - 2021/12
Mauna Loa (MLO)	USA	20 °N, 156 °W	GCMS	1992/12 - 2021/12
Cape Kumukahi (KUM)	USA	20 °N, 155 °W	GCMS	1995/11 - 2021/12
Niwot Ridge (NWR)	USA	40 °N, 106 °W	GCMS, Perseus-GCMS	1992/12 - 2021/12
Barrow (BRW)	USA	71 °N, 157 °W	GCMS	1993/01 - 2021/12
Alert (ALT)	Canada	83 °N, 62 °W	GCMS	1993/02 - 2020/12

Alaska Coast Guard (ACG)	USA	63 °N, -150 °S	Perseus-GCMS	2015/07 - 2017/10
Argyle (AMT)	USA	45 °N, -69 °S	Perseus-GCMS	2015/01 - 2020/12
Boulder Atmospheric Observatory (BAO)	USA	40 °N, -105 °S	Perseus-GCMS	2015/01 - 2016/07
Briggsdale (CAR)	USA	41 °N, -104 °S	Perseus-GCMS	2015/01 - 2020/12
Offshore Cape May (CMA)	USA	39 °N, -74 °S	Perseus-GCMS	2015/01 - 2020/12
CARVE (CRV)	USA	65 °N, -148 °S	Perseus-GCMS	2015/01 - 2020/12
Dahlen (DND)	USA	47 °N, -99 °S	Perseus-GCMS	2015/02 - 2016/11
Estevan Point (ESP)	Canada	49 °N, -126 °S	Perseus-GCMS	2015/01 - 2020/12
East Trout Lake (ETL)	USA	54 °N, -105 °S	Perseus-GCMS	2015/01 - 2020/12
Harvard Forest (HFM)	USA	43 °N, -72 °S	Perseus-GCMS	2016/03 - 2020/08
Homer (HIL)	USA	40 °N, -88 °S	Perseus-GCMS and Perseus-GCMS	2015/01 - 2020/12
INFLUX (INX)	USA	40 °N, -86 °S	Perseus-GCMS and Perseus-GCMS	2015/01 - 2020/12
Park Falls (LEF)	USA	46 °N, -90 °S	Perseus-GCMS	2015/01 - 2020/12
Lewisburg (LEW)	USA	41 °N, -77 °S	Perseus-GCMS	2015/01 - 2020/12
Mt. Bachelor Observatory (MBO)	USA	44 °N, -122 °S	Perseus-GCMS	2015/02 - 2020/12
Marcellus Pennsylvania (MRC)	USA	41 °N, -78 °S	Perseus-GCMS	2015/05 - 2020/12
Mashepee (MSH)	USA	42 °N, -70 °S	Perseus-GCMS	2016/05 - 2020/12
Mt. Wilson Observatory (MWO)	USA	34 °N, -118 °S	Perseus-GCMS	2015/01 - 2020/12
NE Baltimore (NEB)	USA	39 °N, -77 °S	Perseus-GCMS	2018/04 - 2020/12
Offshore Portsmouth (NHA)	USA	43 °N, -70 °S	Perseus-GCMS	2015/01 - 2020/12
NW Baltimore (NWB)	USA	39 °N, -77 °S	Perseus-GCMS	2018/11 - 2020/12
Poker Flat (PFA)	USA	65 °N, -149 °S	Perseus-GCMS	2015/01 - 2020/12
Rarotonga (RTA)	Cook Islands	-21 °N, -160 °S	Perseus-GCMS	2015/01 - 2020/11
Offshote Charleston (SCA)	USA	33 °N, -80 °S	Perseus-GCMS	2015/01 - 2020/12
Beech Island (SCT)	USA	33 °N, -82 °S	Perseus-GCMS	2015/01 - 2020/12

Southern Great Plains (SGP)	USA	37 °N, -98 °S	Perseus-GCMS	2015/01 - 2020/12
Sutro Tower (STR)	USA	38 °N, -122 °S	Perseus-GCMS	2015/01 - 2020/12
Offshore Corpus Christi (TGC)	USA	28 °N, -97 °S	Perseus-GCMS	2015/01 - 2020/12
Trinidad Head (THD)	USA	41 °N, -124 °S	Perseus-GCMS	2015/01 - 2020/12
Thurmont (TMD)	USA	40 °N, -77 °S	Perseus-GCMS	2017/08 - 2020/12
West Branch (WBI)	USA	42 °N, -91 °S	Perseus-GCMS	2015/01 - 2020/12
Walnut Grove (WGC)	USA	38 °N, -121 °S	Perseus-GCMS	2015/01 - 2020/12
Moody (WKT)	USA	31 °N, -97 °S	Perseus-GCMS	2015/01 - 2020/12

Table S2: Measurements used in this work from the NOAA network. Samples used to derive global emissions were collected at the stations ALT, BRW, NWR, KUM, MLO, SMO, CGO and SPO. Data from these stations, along with a few other sites (ESP, THD, PFA and RTA), were also used for deriving background for NOAA inversions. Emissions estimates for the USA were derived from data collected at all North American stations. The measurements were obtained from air collected in flasks that is subsequently analysed in the Boulder, USA, laboratories on a single GCMS. The data period is the periods of data used in this work and not the periods of data availability. CARVE is the Carbon in Arctic Reservoirs Vulnerability Experiment; INFLUX is the Indianapolis Flux Experiment.

Station	Country	Longitude & Latitude	Instrument	Data period
AGAGE				
Mace Head (MHD)	Ireland	53 °N, 10 °W	GCMS-Medusa	2003/11 - 2021/12
			ADS–GCMS	1994/11 - 2004/12
Trinidad Head (THD)	USA	41 °N, 124 °W	GCMS-Medusa	2005/03 - 2021/12
			Archive	1973/10 - 2016/04
Ragged Point (RPB)	Barbados	13 °N, 59 °W	GCMS-Medusa	2005/05 - 2021/12
Cape Matatula (SMO)	American Samoa	14 °S, 171 °W	GCMS-Medusa	2006/05 - 2021/12
Cape Grim (CGO)	Australia	41 °S, 145 °E	GCMS-Medusa	2004/01 - 2021/12
			ADS–GCMS	1998/03 - 2004/12
			Archive	1978/04 - 2011/06
Gosan (GSN)	South Korea	33 °N, 126 °E	GCMS-Medusa	2008/01 - 2020/12
Jungfraujoch (JFJ)	Switzerland	47 °N, 8 °E	GCMS-Medusa	2008/02 - 2020/12
Monte Cimone (CMN)	Italy	44 °N, 10 °E	ADS–GCMS	2012/01 - 2020/12
Tacolneston (TAC)	UK	53 °N, 1 °E	GCMS-Medusa	2012/12 - 2020/12
Taunus (TOB)	Germany	50 °N, 8 °E	GCMS	2013/10 - 2020/12

Table S1. Measurements used in this work. Mole fraction measurements from MHD, THD, RPB, SMO and CGO were used to derive global emissions. Measurements from GSN were used to derive emissions in East Asia and measurements from MHD, JFJ, CMN, TAC and TOB were used to derive emissions in Europe. The data period is the periods of data used in this work and not the periods of data availability. Measurements with the instrument listed as GCMS-Medusa were measured in situ, whereas those listed as Archive were measured with a GCMS-Medusa instrument ex situ.
